# Retraction of “Novel Approach to Photocatalytic Removal of Linezolid by Advanced Nano-Biochar/Bismuth Oxychloride Hybrid”

**DOI:** 10.1021/acsomega.6c07235

**Published:** 2026-07-09

**Authors:** Mahmoud Samy, Ahmed Tawfik, Ahmed I. Osman, Ribh S. Abodlal, Ali El-Dissouky, Tarek E. Khalil, Ehab El-Helow, Mohamed Gar Alalm

The authors retract “Novel Approach to Photocatalytic Removal of Linezolid by Advanced Nano-Biochar/Bismuth Oxychloride Hybrid” (DOI: 10.1021/acsomega.4c04007) to ensure the accuracy of the scientific record following image concerns that affect the reliability of the conclusions.

In Figure 2f and 2g, the EDX patterns for Bi_12_O_17_C_l2_ and biochar/Bi_12_O_17_C_l2_ seem identical, which is not consistent with their intended representation as distinct samples. This reveals uncertainties regarding the assignment of the images and prevents full verification of the reported results. Figure 2f also shows signs of editing.

The two XRD patterns for the material shown in Figure S6 before and after treatment are identical, which raises concerns regarding the attribution and representation of the underlying characterization data.

Taken together, these concerns compromise the integrity of the reported characterization data. As such, this article is being retracted.

The original article was published on July 8, 2024 and retracted on July 9, 2026.

